# Intraocular pressure variation after conventional extracapsular cataract extraction, manual small incision cataract surgery and phacoemulsification in an indigenous black population

**DOI:** 10.11604/pamj.2020.36.119.16942

**Published:** 2020-06-23

**Authors:** Oluwatoyin Helen Onakpoya, Adenike Odunmorayo Adeoye, Bernice Oluwakemi Adegbehingbe, Sarat Abolore Badmus, Bolajoko Abidemi Adewara, Oluwaseun Olaniyi Awe, Patrick Agadaigho Udonwa

**Affiliations:** 1 Department of Ophthalmology, Obafemi Awolowo University, Ile-Ife, Nigeria

**Keywords:** Intraocular pressure, phacoemulsification, extra capsular cataract extraction

## Abstract

**Introduction:**

intraocular pressure changes have been reported following the various cataract surgical technique. This study aims to compare the intra-ocular pressure (IOP) variation following conventional extra-capsular cataract extraction (ECCE), manual small incision cataract surgery (MSICS) and phacoemulsification in an indigenous black population.

**Methods:**

a comparative cross-sectional study of adult patients aged 40 years and above who had pressure was measured with Goldman’s applanation tonometer pre-operatively and 1^st^ day, 1^st^ week, 1^st^ month as well as 3^rd^ month post-operative periods and recorded. Data was analyzed using SPSS version 21. Mean IOP changes between study groups were compared using ANOVA. P-value of < 0.05 was taken as statistically significant.

**Results:**

total of 82 patients consisting of 20(24.4%) ECCE, 32(39%) MSICS and 30(36.6%) phacoemulsification with mean preoperative 13.4mmHg, 13.5mmHg and 14.1 mmHg (p = 0.657) respectively was studied. Mean IOP increased from baseline at 1st day post-operative period in the ECCE and MSICS groups (13.4 ± 3.0mmHg to 13.7 ± 4.5mmHg (p = 0.84) and 13.5 ± 3.1mmHg to 15.3 ± 5.1mmHg (P = 0.48) respectively), and decrease in the phacoemulsification group (14.1 ± 2.6mmHg to 13.9 ± 3.5mmHg (p = 0.378). There was a decline in IOP in all the 3 study groups by one week post-operative period; the difference was significant only in the ECCE group (p = 0.032). By 3^rd^ month postoperatively, there was a reduction in mean IOP when compared with pre-operative IOP the difference being greatest in the ECCE group.

**Conclusion:**

ECCE, MSICS, and Phacoemulsification cause a decline in IOP below preoperative levels at 3^rd^ month postoperatively in the MSICS group.

## Introduction

Cataract is the commonest cause of blindness worldwide, accounting for about 48% of blindness worldwide and a third of the global burden of visual impairment [1, 2]. Cataract surgery is the most performed surgical operation in ophthalmic practice worldwide and in some places, the most common elective surgical procedure [3]. Cataract extraction technique has evolved over the years from Intra-Capsular Cataract Extraction (ICCE) to conventional Extra-Capsular Cataract Extraction (ECCE) through Manual Small Incision Cataract Surgery (MSICS) and Phacoemulsification [4]. Intraocular pressure changes following cataract surgery have been previously reported [5-10]. Both increased and decreased IOP beyond normal levels have been reported to have deleterious effect on the eyes. While hypotony is associated with increased risk of post-operative choroidal effusion or hemorrhage, elevated IOP as well as large IOP fluctuations have been shown to increase the risk of retinal and choroidal ischemia [5]. Sustained elevation of IOP leads to optic nerve head damage. Infact, elevated IOP is a modifiable risk factor in glaucoma; a disease which is one of the leading causes of blindness in Nigeria, and which runs an aggressive course. Previous researchers have reported conflicting results in IOP fluctuations following cataract surgery [6-10]. A transient rise in IOP in the immediate post-operative period in non-glaucomatous eyes following MSICS and conventional ECCE was reported and attributed to the type of viscoelastic material as well as the cataract surgical technique used [10]. Large superior incision in conventional ECCE may cause a distortion in anterior chamber angle structure and influence IOP, which is less likely to occur in MSICS [10]. Other researchers however reported a reduction in IOP following phacoemulsification [11, 12]. This IOP reduction was attributed to the activation of IL-1a and Endothelial leukocyte-adhesion molecule (ELAM)-1 expression by non-glaucomatous trabecular meshwork cells by ultrasound during phacoemulsification thus creating a fluid pressure gradient that exert similar effect as increased IOP [13]. While majority of these studies reported IOP patterns following one or two different cataract techniques, mostly on Caucasians, the need to study an indigenous black population considering the differences in glaucoma pattern, epidemiology, course and complication, cannot be overemphasized. This is particularly important as IOP remains an important factor in the pathophysiology and treatment of glaucoma. This study is designed to determine and compare the IOP changes following conventional ECCE, MSICS, and phacoemulsification in an indigenous black population.

## Methods

This was a comparative cross-sectional study involving consecutive adult patients aged 40 years and above who had uneventful cataract extraction at the Obafemi Awolowo University Teaching Hospitals Complex (OAUTHC) Ile-Ife irrespective of the type. Patients were classified based on the type of cataract surgery they had into 3 groups: the conventional ECCE, MSICS, or phacoemulsification group. Patients with ocular hypertension, glaucoma, pseudo exfoliation, pre-existing trauma, complicated cataract, posterior capsular rent, vitreous loss, hyphema, wound leak, iris prolapse, corneal edema, postoperative uveitis, postoperative endophthalmitis were excluded. Peribulbar anesthesia (2% xylocaine in adrenaline) was used in patients who had conventional ECCE and MSICS, while topical 0.5% tetracaine was used for patients who had phacoemulsification. Conventional ECCE involved raising a fornix based conjunctival flap, and a partial thickness incision was made through about two-thirds depth of anterior limbal area from 10 to 2 oclock. A stab incision into anterior chamber (AC) at 12 oclock position was made and viscoelastic substance was injected into the AC. Anterior capsulotomy, using can-opener method was done and corneoscleral section completed. Hydro dissection, delivery of lens nucleus, and aspiration of the cortex was done. A single piece rigid polymethyl methacrylate intraocular lens (IOL) with a 6.0-mm optic was implanted in the capsular bag after injection of a dispersive viscoelastics. Wound was closed using 10-0 nylon sutures, followed by a meticulous aspiration of viscoelastic substance out of the AC and conjunctival flap was then repositioned. For the MSICS, a 7.5mm self-sealing sclero-corneal tunnel incision 1mm into clear cornea and 1.5 - 2.0mm behind the limbus was made. Internal incision was about 20% larger than the external incision. Contrary to conventional ECCE, suture was not applied to close the self-sealing scleral incision. All other steps were the same as in conventional ECCE. A 3mm self-sealing sclera-corneal incision was made when performing phacoemulsification. A continuous curvilinear capsulorrhexis was done and lens nucleus was emulsified using crack-and-flip technique and aspirated by a phacoemulsifier. A foldable hydrophilic acrylic PC IOL was implanted in the capsular bag. In all 3 groups of patients, a dispersive viscoelastic material was used and meticulously aspirated out of AC after implantation of the IOL. All eyes received sub-conjunctival injection of 2mg dexamethasone and 20mg gentamicin at the end of the surgery. All procedures were carried out under aseptic technique and under Carl Zeiss operating microscope illumination and magnification. Post-operatively, all eyes received topical antibiotics, mydriatics, and steroids for 6-8 weeks. Intraocular pressure was checked pre-operatively, first day post-operative period, one week post-operative, three weeks post-operative, six weeks post-operative, and three months post-operative periods using Goldmann applanation tonometer on Carl Zeiss slit lamp biomicroscope. The age, sex, laterality, and type of cataract surgery were recorded into a predesigned study proforma. The study was carried out in full compliance with the Helsinki declaration on research involving human subjects. The Ethical Committee of OAUTHC, Ile-Ife, approved the study. Data obtained was analyzed using SPSS soft wear version 16. The IOP measurements before and at various time points after surgery across the 3 groups were reported in mean and standard deviation, and IOP changes were compared using ANOVA. Level of statistical significance was set at p value < 0.05.

## Results

**Figure 1:**
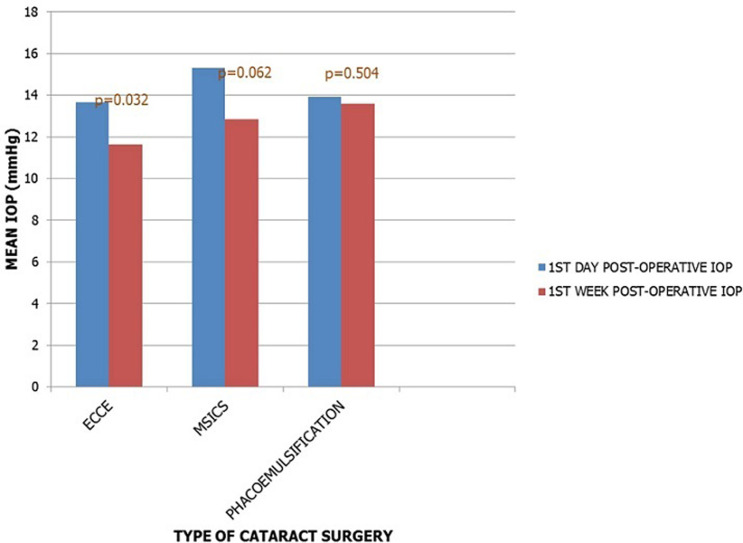
mean intraocular pressure at first day and first week post-operative period in the 3 study groups

**Figure 2:**
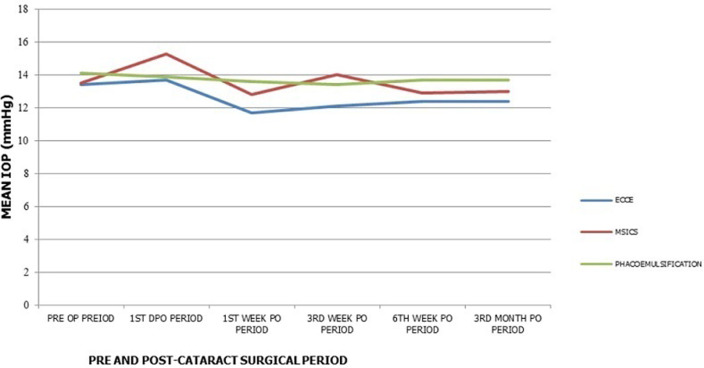
mean intra-ocular pressure pattern following cataract surgery in 82 patients

A total number of 82 patients were studied, of which 20(24.4%) had conventional ECCE, 32(39%) had MSICS, while 30(36.6%) had phacoemulsification in one eye only. The overall mean age of study population was 66.6 ± 11 years. The mean ages for the individual study groups were 63.6 ± 10.1 years for the conventional ECCE group, 63.3 ± 9.2 years for the SICS and 70.8 ± 12.0 years for the phacoemulsification group (Table 1). There was no statistically significant difference in the mean age across all the study groups (p = 0.754). Forty-eight (58.5%) patients were males and 45(54.9%) patients had cataract surgery in the left eye (Table 1). Preoperative IOP ranged 8-21mmHg, 8-21mmHg, and 10-20mmHg, with a mean IOP of 13.4 ± 3.0mmHg, 13.5 ± 3.1mmHg and 14.1 ± 2.6mmHg in conventional ECCE, MSICS and phacoemulsification groups respectively. There was no statistically significant difference in mean pre-operative IOP across the 3 study groups (Table 2). There was an increase in the IOP in the first day post-operative period from baseline in the ECCE and the MSICS groups (p = 0.84 and 0.48 respectively), and a decrease in IOP in the phacoemulsification group from 14.1 ± 2.6mmHg to 13.9 ± 3.5mmHg (p = 0.378) was recorded (Figure 1, Table 3). Intra-ocular pressure decrease was observed in all study groups from 1st day post-operative to 1st week post-operative period (11.7 ± 4.2mmHg, 12.8 ±4.8mmHg, and 13.6 ± 3.0mmHg for ECCE, MSICS and phacoemulsification groups respectively) (Table 3). The difference in the mean IOP between 1^st^ day and 1^st^ week post-operative period was found to be statistically significant only in the ECCE group (p=0.032) (Figure 1). While there was an insignificant increase in IOP from 1^st^ week to third week post-operative periods in the ECCE and MSICS groups (p = 0.77 and 0.174 respectively), a paradoxical insignificant decrease (p = 0.32) in IOP in the phacoemulsification group was observed (Table 3). At 6 weeks post-operative period, an insignificant rise in IOP was noticed in the ECCE and phacoemulsification groups (12.4 ± 2.6mmHg, p = 0.25 and 13.7 ± 2.9mmHg, p = 0.75), while a decline in IOP was noticed in the MSICS group (12.9 ± 3.1mmHg, p = 0.103), with subsequent plateauing of IOP in all the 3 study groups at 3 months post-operative period (12.4 ± 1.1mmHg, 13.0 ± 2.1mmHg and 13.7 ± 4.6mmHg for ECCE, MSICS and phacoemulsification groups respectively) (Figure 2, Table 3). In all study groups there was a decrease in IOP at 3 months from baseline, with ECCE group having the highest mean difference of -1.0 ± 2.9mmHg and phacoemulsification having the least IOP reduction of - 0.4 ± 3.6mmHg. The IOP reduction at 3 months from baseline in all study groups was however not statistically significant (Table 3). None of the study patients had an IOP above 21mmHg throughout the study period.

**Table 1:** characteristics of cataract surgical patients studied

CHARACTERISTICS	ECCE	MSICS	PHACO	TOTAL	P-value
**AGE** (Mean ± SD in years)	63.6±10.1	63.3±9.2	70.8±12.0	66.6±11.0	0.337
**SEX** (N (%))					
MALE	9(11.0)	15(18.3)	10(12.2)	48(58.5)	0.52
FEMALE	11(13.4)	17(20.7)	20(24.4)	34(41.5)	
**LATERALITY** (N (%))					
RIGHT EYE	8(9.8)	9(11.0)	20(24.4)	45(54.9)	0.008
LEFT EYE	12(14.6)	23(28.0)	10(12.2)	37(45.1)	
**TOTAL (%)**	20 (24.4)	32(39)	30(36.6)	82(100)	

N= number of cases; SD= Standard Deviation; ECCE: Conventional Extra-capsular Cataract Extraction; MSICS: Manual Small Incision Cataract Surgery; PHACO: Phacoemulsification

**Table 2:** mean pre-operative intraocular pressures in the different study groups

Pre-operative Mean Intraocular Pressure			
**ECCE**	**MSICS**	**PHACO**	**p-value**
13.4±3.0mmHg	13.5±3.1mmHg	14.1±2.6mmHg	0.652

ECCE: Conventional Extra-capsular Cataract Extraction; MSICS: Manual Small Incision Cataract Surgery; PHACO: Phacoemulsification

**Table 3:** mean post-operative intraocular pressure post cataract surgery in the different study groups

Post-operative Mean Intraocular Pressure (p-value)*			
**Period**	**ECCE**	**MSICS**	**PHACO**
Day 1	13.7±4.5mmHg (p=0.84)	15.3±5.1mmHg (p=0.08)	13.9±3.5mmHg (p=0.86)
1st week	11.7±4.2mmHg (p=1.03)	12.8±4.8mmHg (p=0.52)	13.6±3.0mmHg (p=0.41)
3rd week	12.1±4.1mmHg (p=0.22)	14.0±3.9mmHg (p=0.56)	13.4±2.6mmHg (p=0.11)
6th week	12.4±2.6mmHg (p=1.40)	12.9±3.1mmHg (p=0.45)	13.7±2.9mmHg (p=0.55)
3rd month	12.4±1.1mmHg (p=0.14)	13.0±2.1mmHg (p=0.65)	13.7±4.6mmHg (p=0.75)

(p-value)*-For differences between mean Intraocular Pressure (IOP) in the specific post-operative periods in relation to mean preoperative IOP; ECCE: Conventional Extra-capsular Cataract Extraction; MSICS: Manual Small Incision Cataract Surgery; PHACO: Phacoemulsification

## Discussion

In this study, there was no significant difference in the mean age and pre-operative IOP between the study groups. An increase in mean IOP at 1^st^ day post-operative period from baseline was observed in patients who had conventional ECCE. This was contrary to the study by Kim [7], who reported a decrease in mean IOP in patients who had conventional ECCE. The disparity might have been due to the fact that, in this study, a slightly larger incision size was used when compared to that used by Kim. Furthermore, as opposed to a dispersive viscoelastic material used in this study, a cohesive viscoelastic was used in the study by Kim. Dispersive viscoelastic is more difficult to aspirate out of AC than cohesive viscoelastics [14], which therefore, increases the risk of retention of viscoelastic in AC after cataract surgery and the likelihood of causing a higher immediate post-operative IOP [15]. Also, differences in wound closure techniques may have played a role in decreasing outflow of aqueous through the trabecular meshwork as well as racial differences. The mean IOP at 1st day post-operative period in the MSICS group was also increased from base line. Sengupta *et al*. [16], in a study comparing IOP changes after MSICS versus phacoemulsification also reported a similar increase in IOP at 1st day post-operative period in the MSICS group. However, contrary to this finding, a slight but insignificant decrease in IOP was observed in the phacoemulsification group in this index study. This was similar to result obtained in a study by Rainer *et al*. [17] in patients in whom a dispersive viscoelastic material was used during their cataract surgery. Shingleton *et al*. [11] also observed a decrease IOP in glaucomatous patients, but not in normotensive patients, in whom an increase in IOP was reported. Smaller wound size as well as the activation of IL-1a and Endothelial leukocyte-adhesion molecule (ELAM)-1 expression may have been responsible for the decrease in IOP in the 1st day post-operative period. Some studies have reported a rise in IOP at the 1^st^ day post-operative period in patients who had phacoemulsification [10,11,16]. Differences in sample size and pre-operative IOP may have accounted for the disparity in the observed results. The steady decline in IOP observed in all 3 groups from 1^st^ day to 1^st^ week post-operative period may have been due to resolution of inflammation following post-operative steroid use and clearance of remnant viscoelastic material from the AC. Like in previous studies, patients who had phacoemulsification in this study had a decline in IOP from 1^st^ week to 3^rd^ week post-operative period [11, 16]. On the other hand, IOP was found to increase in both ECCE and MSICS groups contrary to findings in other studies [7, 16]. Mean IOP at 6 weeks and 3 months in all 3 study groups was found to be lower than pre-operative IOP. The relatively lower mean IOP at 3 months observed in this study was in keeping with results from previous studies [7, 12, 16], and this further corroborates the fact that cataract surgery plays a role in increasing aqueous outflow facility in patients with open angles, as well as widens and deepens the AC angle in eyes with narrow angles as reported by previous studies [18, 19]. Some factors other than the type of cataract surgery performed, such as the degree of inflammation, steroid responsiveness among others, could influence IOP changes following cataract surgery. But these could not be accurately analyzed in this study because of its retrospective nature.

## Conclusion

Results from previous studies done on mostly non-black population have shown an inconsistent trend in IOP variation following the different cataract surgical techniques. This study reported that conventional ECCE, MSICS, and Phacoemulsification led to a reduction in the mean IOP at 6 weeks and 3 months after cataract surgery and this reduction being more marked in indigenous blacks who had conventional ECCE.

### What is known about this topic

- Intra-ocular pressure fluctuates following various cataract surgical techniques;
- The trend of IOP variation reported by these studies have been conflicting;
- There is paucity of data on IOP changes in an indigenous black population following cataract surgery.

### What this study adds

- This study reported that conventional ECCE, MSICS, and phacoemulsification led to a reduction in the mean IOP at 6 weeks and 3 months after cataract surgery in an indigenous black population;
- Reduction in IOP was most marked in patients who had conventional ECCE.
